# New York Letter

**Published:** 1893-11

**Authors:** Wm. L. Russell

**Affiliations:** 101 West Ninety-third street; New York


### *CORRESPONDENCE.*

---

### NEW YORK LETTER.

---

NEW YORK, October 15, 1893.

The first meeting of the season was held last Thursday evening. A goodly nnmber of fellows were present. Many of them unusually robust looking and well bronzed from the summer sun.

#### THERAPEUTIC SUGGESTIONS.

The paper of the evening was by Dr. Baruch. He stated that many therapeutic measures, such as venesection and the opium treatment of peritonitis, thoroughly entrenched in the minds of the profession though they seem to be, had fallen from the place formerly occupied by them. At present, too, there were only a few remedies which could be called specifics. In acute diseases it might be said that, generally speaking, medicines were injurious. The importunity of the patient and the friends, for the immediate relief of symptoms, was often now the reason for prescribing medicines. On the other hand, there was also a neglect of new medicinal remedies, some of which were of great value.

Rest was a recognized agent of cure in many conditions, but neglect of applying the principle to inflamed internal organs, by means of diet or vicarious action of other organs, was quite common. This was also true of the treatment of chronic diseases. Intestinal antisepsis in the treatment of typhoid fever he regarded simply as a fad. Not so, however, with the treatment by baths, which had met with astonishing success in Europe for many years. Dr. Loomis regarded Dr. Baruch as an enthusiast in regard to baths and other hydrotherapeutic measures, but believed his statement founded on common sense.

The treatment of pneumonia had gone through many periods but the statistics of seventy-five years at the Massachusetts General

Hospital covering the venesection, antimony, calomel, stimulant, expectant, antipyretic and antiseptic periods, showed an almost even death-rate right through. He thought then that treatment in this instance had thus far proved of little value, and it was quite possible that it was often injurious. As a physician advanced in experience he relied less and less upon drugs and more and more upon hygiene, dietetics, exercise and freedom from disturbing influences. It was well to treat diseases from the etiological standpoint.

Dr. Jacobi believed the science of medicine to embrace diagnosis and therapeutics together, and not either alone. It was the tendency of disease to recover, but medicines were occasionally required. Even if it were only to relieve a symptom, they might be of value. There was often much to be gained by relieving symptoms, as for instance in whooping cough, where complications might thus be avoided. It was true that where the tendency was toward a definite course and recovery, the less medicine given the better, but although whooping cough had such a tendency, it could be shortened by judicious medications. Nor would he despise any remedy, not even venesection, which he thought was the only thing to save certain cases of pneumonia.

Dr. Peabody did not agree with Dr. Baruch's views regarding the treatment of anemia by means of change and fresh air only, and the reflections cast upon the use of iron. Many patients could not get the former, and iron was of unquestionable value. He had some faith in intestinal antisepsis also. If fermentation were prevented, and digestion promoted by it, blood repair was assisted. He thought that all other treatmemt of typhoid fever faded into insignificance in the presence of cold bathing. It could not be afforded by private patients, and he had adopted a modification which he had found of some value. He called it the bed bath. He had the mattress so arranged that it was depressed in the center; it was then covered with a rubber sheet which was elevated at the edges of the bed by means of rolled blankets. The patient was thus made to lie in a puddle of water, which was kept at a temperature of 65° F. by means of ice, and was splashed over him, friction being applied meanwhile, by an attendant. This was not as good a measure as the tub, but was better than the pack. Before any new and radical measure of treatment could be generally adopted, education of the public was necessary.

AN IMPROVED MILK SUPPLY.

This was the subject under consideration by the section in pediatrics, at the last meeting. Although much of the discussion had a local rather than a general bearing, the subject is one of great importance everywhere.

Dr. N. P. Northrup opened the discussion by the startling, but undoubtedly true, statement, that nearly all the milk used in this city was from twenty-four to forty-eight hours old when delivered.

Dr. R. G. Freeman emphasized this by adding that some of it was brought three hundred miles on slow trains, and was forty-eight hours old when delivered.

Dr. Northrup gave an account of the object and work of the Walker Gordon laboratory which has been established in Boston for some time, and has recently opened a branch here. The milk supply of the laboratory is carefully scrutinized. The cows are inspected monthly by a veterinary surgeon. Those having a quiet disposition and a tendency to grow fat if left alone are the most satisfactory. A strictly butter cow is not best; in fact the best cows are those which are best adapted for raising their own calves on their own milk. The alkalinity of the milk is controlled by the character of the feed, and overstimulation of all kind is avoided. The milk is cooled to 45° F. before shipment, and rises to 50° F. on the journey. The cows are milked at 4:30 A. M., and the milk reaches the laboratory at 8 : 15 A. M. The greatest care is taken to secure its cleanliness.

The object of the laboratory is to modify the milk, so as to bring it into closer harmony with the constitution of human milk. It is first placed in a centrifugal machine, and the grosser foreign matter thus removed. The composition of an average cow's milk is taken as representing in solid matter four per cent. each of fat, milk-sugar and albuminoids. These proportions are then changed to four per cent. fat, seven sugar and two albuminoids, to represent a "heavy" breast milk, and to three per cent. fat, six milk-sugar and one albuminoids to represent a "light" breast milk. This, he said, was much more scientific than the ordinary dilution of cow's milk, whereby all the constituents were diluted equally. One-sixteenth part of lime water was added to the milk to insure alkalinity. The greatest objection to the plan was its expense.

Dr. Coit, of Newark, N. J., described the means of securing a proper milk supply which had been adopted by a number of physicians in the county in which he lived. He said that the public was lax and indifferent in regard to the matter, and even the profession was hardly as active as it ought to be. Reform in dairy methods was badly needed, in regard to the breeding of the cows, their care, feeding, and quarters, and the methods of preserving and transporting the milk. In thirty per cent. of the dairies one or more tubercular cows were to be found. The plan adopted by them consisted first in the appointment of a medical commission. These served without pecuniary reward, and were representative men. They had been fortunate enough to find a model dairy twelve miles from Newark, and had made a hard and fast contract with the owner in regard to all matters pertaining to securing a pure milk. In return they were to give him all the support possible regarding price and patronage. The building in which the cows were kept was of stone, and contained nothing but the cows, no other live stock being allowed nearer than three hundred yards. Light, ventilation, and the comfort of the cows were carefully provided for. No phenomenal milkers were desired, and kind treatment was insisted upon. The floor of the building was of cement, and allowed of flushing. The cows were groomed twice daily, and the milkers were required to wash their hands, and the udders of the cows, before beginning to milk. The cans were specially constructed, so that the milk entered through a sieve, and no dust could enter. As soon as filled, each can was closed tightly. The milk was then removed to a refrigerator chamber, which was not entered while it contained any milk. It was strained as it entered, and as it left after cooling was received in bottles. This milk had kept without further treatment for eleven days, at ordinary temperature.

Dr. Koplik believed that while a milk laboratory might be very interesting, it could never be of much practical benefit to those suffering most from impure milk, because of the expense. Milk that had not reached the stage of incubation, that is, the stage just before it turned sour, was safe. Cow's milk, however, not only contained casein in excess, but the character of the albuminoid was somewhat different than in human milk. Large hard curds were likely to form in the child's stomach. This could only be avoided by proper dilution. Sterilization was the most valuable innovation regarding the management of milk, yet introduced. He thought that a good milk, properly diluted, and sterilized, met the problem of the thousands, almost any reasonable plan working well enough for a few cases. Sterilization should be done at as low a temperature as possible, and only sufficient for twenty-four hours should be prepared. Transportation of sterilized milk was not advisable, as the fat was liable to separate in globules.

### HELMHOLTZ.

Among the distinguished scientists who have visited New York this year none aroused more interest than the illustrious inventor of the ophthalmoscope. The lecture room of the College of Physicians and Surgeons, where he made a short address to the students, was packed to the doors, and many were unable to get in. Prof. von Helmholtz is no longer young, but has the robust appearance and active manner of vigorous old age. He told in a simple, informal way, the story of the invention of the ophthalmoscope. He was at the time a young teacher of physiology, but had studied physics thoroughly, with the idea of devoting himself to that subject. He was deterred from carrying out his desire by financial necessities. As a teacher of physiology, it devolved upon him to attempt to explain the illumination of animal's eyes which was noticed in a darkened room. He knew also that the eyes of people sometimes presented a similar phenomenon, and that a student in Vienna had even got a faint glimpse of the interior of the eye of a fellow-student, by the light reflected from the spectacles he was wearing. By carefully applying the laws of optics to these phenomena, he was led to invent the instrument, which has been of such value to medical science. He closed by urging his hearers to pursue their meditations carefully to their logical conclusion. The reception given by the students was such as can come only from college students.

101 *West Ninety-third street.* WM. L. RUSSELL.

